# Deep Structure Usage of Electronic Patient Records: Enhancing the Influence of Nurses' Professional Commitment to Decrease Turnover Intention: Deep Structure Usage and Turnover

**DOI:** 10.1155/2024/5822368

**Published:** 2024-11-11

**Authors:** Hao-Yuan Chang, Guan-Ling Huang, Yea-Ing Lotus Shyu, Alice May-Kuen Wong, Shih-I Tai, T. C. E. Cheng, Ching-I Teng

**Affiliations:** ^1^School of Nursing, College of Medicine, National Taiwan University, Taipei, Taiwan; ^2^Department of Second Degree Bachelor of Science in Nursing, College of Medicine, National Taiwan University, Taipei, Taiwan; ^3^Department of Nursing, National Taiwan University Hospital, Taipei, Taiwan; ^4^Graduate Institute of Management, Chang Gung University, Taoyuan, Taiwan; ^5^School of Nursing, Chang Gung University, Taoyuan, Taiwan; ^6^Department of Physical Medicine and Rehabilitation, Chang Gung Memorial Hospital, Linkou, Taoyuan, Taiwan; ^7^Business Administration, Management, Faculty of Business, Department of Logistics and Maritime Studies, The Hong Kong Polytechnic University, Hong Kong, China; ^8^Department of Business and Management, Ming Chi University of Technology, Taishan, Taiwan

**Keywords:** electronic patient records, nurse, professional commitment, regression, survey, system usage, turnover intention, workforce

## Abstract

**Background:** Organizational turnover exacerbates the shortage of nurses in the global workforce. However, no study has yet explored how deep structure usage—nurses' integration of electronic patient records into nursing practice delivery—reduces their turnover intention and moderates the impact of affective, continuance, and normative professional commitment on their turnover intention.

**Aims:** To ascertain (1) the linkage between the deep structure usage of electronic patient records and nurses' organizational turnover intention and (2) the moderating role of deep structure usage on the associations between elements of commitment (affective, continuance, and normative) and turnover intention.

**Methods:** Using a cross-sectional survey and proportionate random sampling by ward unit, we collected data from 417 full-time nurses via a self-administered questionnaire. We performed hierarchical regression analyses to test the study hypotheses.

**Results:** Deep structure usage was not directly related to organizational turnover intention (*β* = −0.07, *p*=0.06). However, the results suggested that deep structure usage may enhance the effect of high affective commitment on nurses' organizational turnover intention (*β* = −0.09, *p*=0.04), while potentially mitigating the effect of low continuance commitment on organizational turnover intention (*β* = 0.10, *p*=0.01).

**Conclusions:** Deep structure usage of electronic patient records helps to ease nurses' workload and facilitates their retention, which is particularly due to their affective commitment (attachment) but not their continuance commitment (switching costs).

**Implications for Nursing Management:** Nursing management may advise hospital management that medical records systems need to be improved and fully embedded for nursing care delivery, as a more in-depth use of these systems can help to retain nurses.

## 1. Introduction

The worldwide shortage of nurses is a serious issue in healthcare management [1]. The shortfall is estimated to reach 10 million by 2030 [2], and the demand for nurses is expected to continue to increase [3]. A shortage of nurses may lead to heavy workloads and stress for nurses and consequently reduce care quality [1]. Due to the fact that the shortage may result from nurses' decisions to leave a hospital [4], it is important to understand the reasons for nurses' organizational turnover intention.

The literature has examined many sources of nurses' turnover intention, such as rising incivility [5], high levels of emotional labor [6], a lack of authentic leadership [7], high job demands [8], and low levels of professional commitment [9]. Among these, professional commitment helps retain nurses [9]. Professional commitment likely reduces nurses' organizational turnover intention when nurses' sense of professionalism binds them to an organization.

Nurses' professional commitment facilitates innovative behavior [10], for example, active use of technology; therefore, professional commitment may change the effectiveness of technology use. Hence, we believe that the use of technology interacts with professional commitment to affect nurses' turnover intention. However, the literature has not yet examined how technology may moderate the influence of professional commitment and its dimensions on nurses' turnover intention, thus indicating a research gap. Technology in the healthcare context has aided nurses' work engagement and improved care outcomes [11, 12], although not all studies have found positive aspects of technology introduction. For example, poorly designed electronic medical records systems impose pressure on nurses [13]. Hence, it is reasonable to expect that technology can change the impact of professional commitment on nurses' turnover intention, which warrants further research to address this gap.

Research resolving this gap is important from both academic and practical standpoints. Academically, such research can furnish new knowledge on nursing management by introducing technological influences. Practically, such research can offer new insights for multiple parties—such as nurse managers, technology developers, and policy-makers—by informing them how to leverage technology in nursing workplaces.

To address this research gap, we needed to choose a focal system. Electronic patient records systems are prevalent in the healthcare context. This widespread prevalence motivated us to select electronic patient records systems as a representative technology for investigation. There are many types or levels of system use [14]. Among them, the deep structure usage of information is key to positive outcomes. Also, we postulate that the deep structure usage of electronic patient records is a candidate moderator that likely moderates the impact of professional commitment on nurses' turnover intention and has a direct effect on nurses' turnover intention. For example, deep structure usage reflects nurses' use of the electronic medical records system to understand patients' health trajectories (such as changes in critical biological indices), thus facilitating nurses' preparation and implementation of professional nursing care plans. In contrast, likely due to the suboptimal design of a system or other reasons, nurses not employing deep structure usage need to ask patients to retrieve information from memory, check whether there are any other records or paper copies, or make decisions without such information. None of these practices is good for patient care; therefore, they may affect nurses' perceptions of organizational workplaces, hence affecting their retention decisions.

## 2. Background

Nurses' workplaces are characterized by work stress and time pressure, which may directly prompt them to leave their organization or profession. Both of these factors have aggravated the nursing shortage [4]. To retain nurses, it is important to consider professional commitment, which has been defined as a “psychological link between an individual and the decision to continue in an occupation, career or profession” [9], p.2). The extant literature has identified professional commitment as an important factor in decreasing nurses' professional and organizational turnover intention [9].

Commitment to a profession or career may be cultivated by extensive education [15]. However, previous studies have not examined how technology may help to amplify the protective effects of professional commitment on nurses' turnover intention, thus demonstrating a knowledge gap. Research addressing this is important because it can help in the design of various technologies that are introduced to nursing workplaces—such as electronic health records [11] and mobile robots [12]—to reduce nurses' turnover intention. Many systems are used in nursing workplaces, and one of the most widely used is the electronic patient records system, which collects the information necessary to provide nursing care [16]. In many countries, electronic patient records systems are mandatory for forming and maintaining an overview of patients [17]. Therefore, these systems are suitable for our research into how their use can affect the influence of nurses' professional commitment on their turnover intention.  Table 1 lists the definitions of the study concepts.

Deep structure usage has been defined as “the use of the system for the task” [14], p. 235) and the use of a system to understand and improve patients' health [14]. Hence, we define deep structure usage as the integration of patient information from electronic medical records systems into nursing practice delivery. Deep structure usage can enhance users' performance via exploitation (of system functions) and exploration (of novel means of performing activities) [18], thus justifying its inclusion in our research.

**Figure 1:**
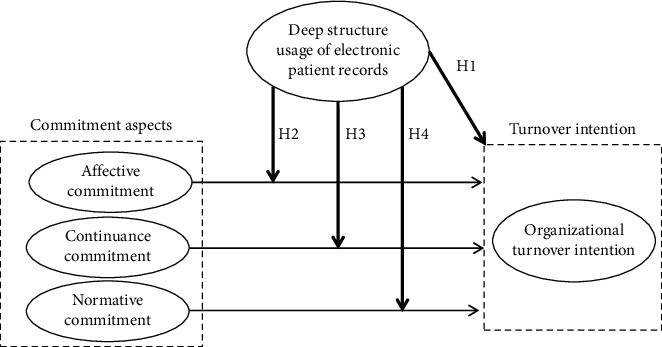
Research framework.

Figure 1 illustrates the research framework. We did not develop hypotheses on the paths from professional commitment dimensions to nurses' turnover intention, as these were examined in previous studies. Thus, we focused on the new concept of *deep structure usage of electronic patient records* and its moderating effects on these pathways. Accordingly, all of the hypotheses in our study are newly introduced to the literature. We discuss the control variables in the Method section and report the test results in the Results section.

Increased deep structure usage of electronic patient records signals that nurses may have a sound knowledge of and an ability to use electronic medical records systems. Thus, nurses are well informed about their patients' statuses and are able to provide quality care [19]. Quality care is positively associated with team effectiveness [7], which enhances nurses' job satisfaction and organizational retention [20]. Moreover, enhanced job satisfaction indicates that nurses perceive a better working environment, which decreases the risk of burnout and negates turnover intention [7, 21]. Hence, we hypothesize:

H1 The deep structure usage of electronic patient records is negatively related to organizational turnover intention.
  Affective commitment indicates the degree to which nurses feel emotionally attached to their organization [10], reflecting that nurses have a high degree of trust in organizational systems and a strong relationship with the organization, which is defined as job embeddedness [22]. Such emotional attachment and trust should reduce nurses' turnover intention. At nursing workplaces, digital systems enable greater consolidation of medical records to facilitate care processes [16], thus deepening nurses' trust in organizational systems and their relationships with the organizations, i.e., increasing job embeddedness. Accordingly, deep structure usage enhances the relation between high affective commitment and reduced organizational turnover intention. Conversely, nurses with high affective commitment but with limited access to deep structure usage may not easily build deepened trust and relationships with the organization, i.e., limited job embeddedness. Hence, we hypothesize:
H2 The deep structure usage of electronic patient records enhances the relation between high affective commitment and reduced organizational turnover intention.
  The deep structure usage of electronic patient records indicates that nurses use electronic medical records systems to track care activities and ensure patient safety. Such use can help nurses better utilize patient information to enhance the quality of nurse–patient interactions and communication [11], thus helping nurses to complete their tasks. In this case, deep structure usage promotes task-oriented communication and advanced tasks [11], thus achieving good job performance, which increases the perceived benefits of staying with the organization that provides an excellent electronic patient records system. Moreover, deep structure usage can improve nurses' job performance and reduce job-related stress, enhancing the perceived benefits of staying with the organization and thereby reducing their turnover intention. That is, deep structure usage should be more impactful among nurses with strong continuance commitment. Conversely, nurses with high continuance commitment but with limited access to deep structure usage may not experience a change in performance. Hence, deep structure usage could enhance the positive relation between high continuance commitment and reduced turnover intention. Hence, we hypothesize:
H3 The deep structure usage of electronic patient records enhances the relation between high continuance commitment and reduced organizational turnover intention.
  Normative commitment can enhance the quality of care because nurses who possess normative commitment are dedicated to fulfilling their responsibility to provide high-quality care [9]. The provision of quality care is related to job satisfaction [23], which in turn reduces organizational turnover intention [24]. Hence, nurses with strong normative commitment are less likely to harbor organizational turnover intention due to the positive relationship between quality care provision and job satisfaction. The deep structure usage of electronic patient records facilitates nurses' ability to provide high-quality care [19]. Therefore, nurses with normative commitment and access to deep structure usage can deliver superior care compared to those with limited deep structure usage, leading to increased job satisfaction and decreased turnover intention. Conversely, nurses with normative commitment but with limited access to deep structure usage may struggle to provide high-quality care despite their commitment, which can undermine their job satisfaction. As a result, nurses with high normative commitment but with limited deep structure usage may experience increased turnover intention due to their inability to meet care standards, even though they are dedicated to their responsibilities. According to the above inference, it is hypothesized that deep structure usage moderates the relationship between normative commitment and organizational turnover intention, where the presence of deep structure usage enhances the positive effects of normative commitment on reducing turnover intention.
H4 The deep structure usage of electronic patient records enhances the relation between high normative commitment and reduced organizational turnover intention.

The purpose of this study is to examine how deep structure usage of electronic patient records is associated with nurses' turnover intention and how it moderates the association between dimensions of professional commitment and nurses' turnover intention.

## 3. Methods

### 3.1. Design

By adopting a cross-sectional design, we collected data in February 2022 for one month.

### 3.2. Sample and Data Collection

The chosen research site was a large medical center (hospital) in Taoyuan, a major city in northern Taiwan. This hospital is known for its large number of beds and large patient throughput, operating efficiency, advanced medical equipment, and strong support for technology implementation. Hence, the system of electronic patient records was well integrated throughout the hospital.

Eligible nurses were those who were registered and worked full time. Nurses who were supervisors, students, interns, or advanced practice nurses were excluded from the study to increase the compatibility of the sampled nurses. The sampling method involved proportional random sampling, which is known for its randomness, as well as for ensuring a representative sample. From each work unit, we sampled the same proportion of eligible nurses. Hence, it was guaranteed that each work unit, regardless of its staffing complement, had some nurses who were sampled, which may not always be the outcome in simple random sampling.

In total, the sampling frame included 1646 nurses, and we were permitted to make contact with 450 nurses. Hence, the percentage of randomly sampled nurses in each unit was 27% (450/1646 = 27%). Nurses were sampled from 75 units, as allowed. Randomization was performed using computer-generated random digits.

In total, 450 nurses were approached in person by the research assistants. Hard-copy versions of the questionnaire were passed to the participants, and the completed forms were returned directly to the investigators, without passing through the hands of other hospital staff. Among them, 417 participated throughout the study, and their responses were determined to be valid and were used for further analyses. The nonresponse proportion was small (33/450 = 7%). This small proportion suggests that nonresponse bias is likely insignificant. We performed a power analysis to estimate the required sample size. We used the website https://www.analyticscalculators.com/calculator.aspx?id=1, setting a confidence level of 0.95, 10 predictors, a statistical power of 0.80, and an estimated effect size of 0.10, thus resulting in a required size of 172. To increase the testing power, we increased it to 450. The final sample size was 417, which was larger than the estimated sample size of 172.

## 4. Ethical Considerations

The ethical aspects of this study were reviewed and approved (202100275A3C601) by the institutional review board (IRB) of the hospital from which the data were collected. The ethical aspects were further ensured via several methods during implementation: (1) research assistants were recruited from outside the hospital to ensure that nurses' participation in this study was voluntary; (2) research assistants received ethical training before data collection; (3) prospective participants were briefed on the research purpose before consenting to their participation, which involved a written informed consent process; (4) participants were given 3 days to complete the questionnaire to reduce disturbance to their patient care routines; and (5) participants who completed the survey were offered a gift certificate as an expression of the investigators' gratitude.

### 4.1. Measurement

Scale selection was based on the sound reliability and validity of the original scale. We adapted the six items from the subscale “Deep Structure Usage” in *Mindfulness in Information Technology Use* [25] to measure the deep structure usage of electronic patient records, which demonstrated sufficient reliability (*α* > 0.74) and both convergent and discriminant validity [25], their Table 2). We specified the electronic medical records system as the target to assess, and modified the descriptions of the functions to conform to an electronic medical records system; specifically, we modified “calculations on my data” to “care evaluation” to measure the deep structure usage of electronic patient records. Moreover, the three elements of commitment were measured using the scales of Meyer, Allen, and Smith [26]; with six items evaluating affective commitment, four measuring continuance commitment, and four gauging normative commitment. Those items were used and verified by Huang et al. [27] as fulfilling various criteria for indices assessing reliability and validity, including composite reliability (CR), average variance extracted (AVE), indicator loadings, and whether the square roots of the AVE values were greater than the associated correlations. The sufficient performance of all of these indices demonstrated their applicability.

Furthermore, we adopted three items from Chang et al. [24] to measure turnover intention by changing their targets to “the hospital.” The items assessing retention intention were reversely coded to ensure that larger values represented stronger turnover intention. These items exhibited a reliability > 0.70 and good validity in Chang et al. [24]; including loadings > 0.88. The scales of our study had a five-point response option, with 1 representing “strongly disagree,” 2 representing “disagree,” 3 representing “neutral,” 4 representing “agree,” and 5 representing “strongly agree.” We collected participant information on gender, age, and education as control variables. The Appendix lists the study items.

Regarding instrument language, both instruments from Huang et al. [27] and Chang et al. [24] were in traditional Chinese, as used by the nursing population in the present study. The instrument used by Thatcher et al. [25] was in English. However, we adapted their scale to fit our research context. During the process, we consulted senior nurses and experienced nursing scholars to substantiate the contextual features as key words. This substantiated scale was in traditional Chinese, as used in the study population.

### 4.2. Data Analysis

We tested reliability (such as Cronbach's alpha) and validity by using confirmatory factor analysis (CFA). Harman's test was performed to test common method variance (CMV). The descriptive statistics and correlations were calculated. We used SPSS (version 17.0; IBM Corp) to conduct regression analyses. We included gender, age, and education as control variables. The dependent variable was organizational turnover intention. The independent factors included commitment aspects. The moderator involved deep structure usage of electronic patient records. Before testing the moderating effects, the data were mean-centered to avoid multicollinearity issues.

## 5. Results

### 5.1. Demographic Characteristics of Participants

All 417 recruited nurses were full-time registered nurses in a large hospital in Taiwan. Most were female (98.3%), aged between 20 and 49 years of age (96.4%), and had attended universities or colleges (83.7%). The gender composition of the respondents was similar to that of the nurse population (96% female, [28].  Table 3 summarizes the profile of our participants.

### 5.2. Psychometric Properties

We evaluated reliability and validity by using CFA with five factors.  Table 4 lists the analytical results, including the reliability and validity statistics. The Cronbach's *α* values of our study concepts were between 0.76 and 0.94 (> 0.70). All of the Cronbach's *α* values and their lower bounds of the confidence intervals exceeded 0.70, thus indicating “confident” reliability. All of the items of each construct had a CR score of close to (0.69) or greater than 0.70, thus demonstrating sufficient reliability.

### 5.3. Testing Common Method Variance

Most of the indicator loadings (as the *λ* values in the last column of the Table 2) exceeded 0.50, indicating convergent validity. In Table 4, the maximum correlation (0.43) was smaller than the minimum square root of the AVE value (0.66), indicating discriminant validity. All of the correlations in Table 4 were below 0.70. The data in our study performed well in terms of the fit indices in CFA (CFI = 0.90, IFI = 0.91). We tested CMV using Harman's single factor test. Specifically, we used exploratory factor analysis and the Varimax rotation method to determine how the first factor (assumed to be the common method) explained CMV. The value of the first factor was 28.0%, which was lower than the threshold value of 50%; specifically, CMV was unlikely to bias our measurements. We further used the correlation adjustment method [29] to assess the effect of CMV. After removing CMV from the correlations, all of the significant correlations remained significant, thus indicating that CMV is unlikely to bias the findings.

## 6. Hypothesis Testing

We applied the hierarchical regression method to test the hypotheses. The first Model (M1) included the demographics, the second Model (M2) further included the main effect terms, and the third Model (M3) also included the interaction terms.  Table 5 shows the analysis results.

**Figure 2:**
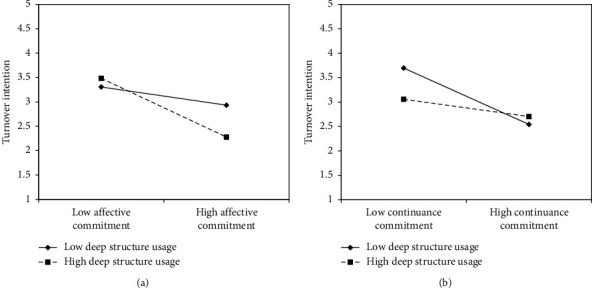
(a) Interactive effect of affective commitment and deep structure usage on organizational turnover intention. (b) Interactive effect of continuance commitment and deep structure usage on organizational turnover intention.

Figures 2(a) and 2(b) illustrate the interactive effects. When affective commitment is high, nurses' turnover intention decreases. Also, when nurses engage in the in-depth use of electronic patient records alongside high affective commitment, their turnover intention is even lower compared to their counterparts. In other words, Figures 2(a) suggests that deep structure usage may enhance the positive effect of high affective commitment on turnover intention. When continuance commitment is low, nurses' turnover intention increases. However, when nurses engage in the in-depth use of electronic patient records, their turnover intention becomes significantly lower than that of their counterparts. In other words, Figure 2(b) demonstrates that deep structure usage may mitigate the negative effect of low continuance commitment on turnover intention. That is, deep structure usage moderates the relationship between continuance commitment and turnover intention in a way that reduces turnover intention.

Most of the regression results supported the study hypotheses. Specifically, deep structure usage of electronic patient records was not associated with organizational turnover intention (*β* = −0.07, *p*=0.06); specifically, it did not support H1. For the moderating effects, deep structure usage *enhanced* the relation between high affective commitment and reduced organizational turnover intention (*β* = −0.09; *p*=0.04), thus supporting H2. Deep structure usage *reduced* the negative relation between continuance commitment and organizational turnover intention (*β* = 0.10, *p*=0.01), which was contrary to the prediction of H3. Moreover, H4 was also not supported (*β* = −0.03; *p*=0.30). We discuss the plausible reasons in the next section.

Our full regression models explained 25% of the variance in organizational turnover intention. These magnitudes could be regarded as having large effect sizes [30]. Moreover, the variance inflation factors were all below 1.40, which signified a lack of multicollinearity [31].

### 6.1. Additional Analyses

#### 6.1.1. Effects of Professional Commitment Elements

Nurse manager may want to know which elements of professional commitment may work best to retain nurses. Hence, we compared the effects of the elements of professional commitment on turnover intention. We found that affective and continuance commitment had stronger effects than normative commitment (*p* < 0.001) on organizational turnover intention.

#### 6.1.2. Effects of Control Variables

Regarding the control variables, we found that age significantly predicts reduced turnover intention (*p* < 0.001). This finding is consistent with previous findings that age is a predictor of actual turnover [32]. This effect may be due to increased responsibility and the difficulty of switching. However, this result needs to be tested in further research.

#### 6.1.3. Effects of Including or Excluding Control Variables

The results may depend on the included control variables. Hence, we removed the control variables from the tested regressions. Such removal did not change any of the testing results (the significant results remained significant).

## 7. Discussion

We found that deep structure usage enhances the positive effect of high affective commitment on turnover intention, while mitigating the negative effect of low continuance commitment on turnover intention. The reason may be that deep structure usage represents nurses' integration of patient information from electronic medical records systems into nursing practice delivery, thus further connecting nurses to their jobs (i.e., enhancing their job embeddedness) [22]. Enhanced job embeddedness should be desirable for nurses who are affectively committed (emotionally attached) to the nursing profession. Hence, deep structure usage could enhance the positive effect of high affective commitment on nurses' turnover intention. The empirical results also support this prediction. The key points of this study are: (1) deep structure usage can moderate the effects of commitment on turnover intention and (2) such moderating effects varied between affective and continuance commitment.

Thus, nurse managers could inform hospital managers of the importance of improving electronic medical records systems to better empower nurses to understand and improve patients' health, thus helping medical facilities to retain highly committed nurses.

## 8. Implications for Theory

### 8.1. Deep Structure Usage

The first contribution of our study is to identify the moderating effect of deep structure usage on the relationship between commitment and turnover intention. Previous research on deep structure usage indicated that it can facilitate team coordination and enhance patient outcomes [33]. However, our study is novel in revealing that deep structure usage of electronic patient records can help moderate (enhance) the protective effect of commitment on turnover intention.

### 8.2. Professional Commitment

The second contribution of our study is to demonstrate that the moderating effects of deep structure usage differ between affective and continuance commitment. Compared with nursing literature on professional commitment [9], our findings clarify that technology use (deep structure usage of electronic patient records) may change the effects of different aspects of commitment.

### 8.3. Nurse Turnover

Our findings indicate that use of an electronic medical records system could help to retain nursing professionals. In deep structure usage, nurses integrate electronic medical records systems with the delivery of care [34]. Most nurses have a positive experience of using records from electronic medical records systems [35], as such systems can help nurses to complete their tasks, thus increasing their job satisfaction, which correspondingly aids their retention [36]. Furthermore, we innovatively found that a useful electronic medical records system may also enhance the effect of affective professional commitment on nurses' retention in an organization.

### 8.4. Nonsupported Hypotheses

The deep structure usage of electronic patient records did not reduce organizational turnover intention, thus not supporting H1. The reason for this effect may be that the use of a system is simply a part of work within an organization, thus exhibiting merely a small (but insignificant) effect. Interestingly, deep structure usage mitigates (but does not enhance) the relation between high continuance commitment and reduced organizational turnover intention, contrary to our prediction in H3. We speculate that the reason for this effect may be that deep structure usage offers value by enabling a user to more deeply understand a process [37], which may potentially increase the individual's confidence in finding a comparable job. This confidence may offset the negative relation between switching costs (continuance commitment) and organizational turnover intention.

Methodologically, moderation is an interaction but does not require the main effect to be moderated to be significant [38]. Hence, we further examined the moderating effect of deep structure usage on the influence of normative commitment on organizational turnover intention; however, we did not find evidence to support H4. We speculate that normative commitment may increase the tolerance of using imperfect electronic patient record systems. Specifically, normative commitment induces nurses to fulfill their perceived obligations by implementing care practices in the nursing profession, whether or not electronic patient record systems are highly useful for patient care. Similarly, normative commitment also imposes a responsibility on nurses to make efforts toward patient care [27]. This responsibility is inconsistent with a nurse's decision to leave a nursing job. Thus, whether or not systems are good does not affect the relation between normative commitment and the turnover intention to leave the organization, thus explaining the lack of support for H4.

## 9. Implications for Practice

We found that deep structure usage of electronic patient records was important because of its moderating effect on retaining highly (affectively) committed nurses. Hence, we recommend that nurse managers advise hospital management to consider investing more resources in electronic medical records systems for better and easier use, for example, by providing training courses at convenient times for nurses to learn more about the novel functions of the systems and by asking nurses to offer suggestions in the developmental stage of the systems [35]. These initiatives could enhance nurses' integration of the systems into their nursing care delivery (deep structure usage) in the hope that such systems, if well designed and maintained, could help retain nurses.

Our findings also offer insights for nurse managers to design strategies to retain nurses in hospitals. Specifically, we found that affective commitment and continuance commitment are negatively related to organizational turnover intention. To enhance nurses' affective commitment, nurse managers could hold monthly campaigns to invite the best-performing nurses to share their best practices, either in professional care routines or in interpersonal interactions with patients and their families. The dissemination of best practices can help in achieving better patient outcomes [39]. Hence, nurses with the best work performance could receive commendations, which may enhance their affective commitment. To enhance nurses' continuance commitment, policy-makers could increase nurses' switching costs by offering additional annual leave, promotions, or perks based on nursing tenure.

We also found that age was a significant predictor of turnover intention. In the age range of our participants, age may be related to reduced family responsibility, thus offering more freedom to quit a job. Hence, nurse managers could invite the relatively young nurses to offer their innovative thoughts for workplace improvement, such as by encouraging them to make suggestions on improvements to the electronic medical records system and assigning them as the key individuals to follow up on such improvements. Such methods may increase nurses' work engagement and reduce their turnover intention. Moreover, hospital management could provide subsidies to young nurses for professional development, thus instilling the sense that the hospital supports their career growth.

### 9.1. Limitations

Our findings were obtained from a single medical center, and this may limit the generalizability of the research results. However, our findings can be applied to other hospitals with similar features to our research site, such as hospitals with many medical departments. Moreover, our model did not control for the clustering effect, which is a limitation. The reason for this lack of control is that we adopted proportionate random sampling. In so doing, each possible nurse/individual received the same opportunity to be approached in any unit, whereas we approached all of the accessible units in the hospital, thus negating the bias due to clustering effects [40].

Our study focused on electronic patient records. As one study cannot easily include many systems, future studies could include other systems (such as automatic material transportation vehicles and in-hospital instant messaging systems) and examine whether such systems have varied effects on nursing workplaces. Moreover, future studies could examine how novel technology such as artificial intelligence or robots, may assist nurses or add stressors to nurses, thus offering enhanced insights into nurse–technology interactions.

Our study aimed to verify that common method bias was not statistically significant. To fully negate such biases, future studies could use data from different sources. For example, they could use nurses' actual turnover records as an alternative data source. Also, our model did not fully explain the variance in nurses' turnover intention. The reason for this effect may involve the complexity of nurses' turnover intention, thus indicating both the difficulties and the opportunities for future studies to fully explain such intentions. Previous studies have explained 35% of the variance in turnover intention in Jiang et al. [5] and 17%–25% in Chang et al. [32]. Our study explained 24%–25% of the variance, which is consistent with previous studies. Future studies may also include the most well-known factors (such as job satisfaction) to increase the explained variance of nurses' turnover intention.

## 10. Conclusions

This study contributed a new concept of *deep structure usage of electronic patient records* in the nursing context. The main message of this study is that the deep structure usage of electronic patient records may help to retain nurses, particularly nurses with strong affective commitment. Prior to this research, systems may have been viewed merely as tools to assist in the completion tasks. However, this research uniquely indicates that well-designed systems may help to fulfill nurses' desire to competently provide well-informed nursing care, thus contributing insights for retaining highly committed nurses. Accordingly, hospital management could invest in well-designed and fine-tuned systems to meet workplace and nurses' needs. Future research could address these limitations noted above by exploring the importance of the deep structure usage of other information systems in nursing workplaces.

## Figures and Tables

**Table 1:** Definitions of the study concepts.

Study concept	Definition
Affective commitment	Nurses' passion for and identification with their profession
Continuance commitment	Nurses' commitment made to their profession because they feel they need to, considering the profit associated with continued participation and the cost associated with leaving
Normative commitment	Nurse-perceived responsibility and loyalty to remain in their profession
Deep structure usage of electronic patient records	Nurses' integration of patient information from electronic medical records systems into nursing practice delivery
Organizational turnover intention	Nurses' inclination to quit their job in a hospital

**Table 2:** Scales and items.

Construct	Item	*M*	SD	*λ*
Affective commitment	The nursing profession is important to my self-image	3.97	0.84	0.65
	I regret entering the nursing profession⁣^∗^	3.91	0.93	0.73
	I am proud that I am in the nursing profession	3.68	0.79	0.76
	I do not like being a nurse⁣^∗^	4.07	0.87	0.78
	I do not identify with the nursing profession⁣^∗^	4.34	0.79	0.67
	I have a passion for the nursing profession	3.41	0.82	0.79

Continuance commitment	Since I have invested so much in the nursing profession, it would be impossible to leave it now	3.23	1.02	0.59
	It would be difficult for me to switch to another profession now	3.41	1.01	0.80
	If I switched to another profession now, my life would be greatly impacted	3.08	1.08	0.82
	The cost is too high to change my profession now	3.36	1.06	0.85

Normative commitment	I consider that those educated in nursing have the responsibility to stay in the profession for a period of time	3.15	1.11	0.66
	I have a responsibility to stay in the nursing profession	3.33	0.94	0.85
	If I left the nursing profession, I would feel guilty	2.18	0.97	0.49
	I stay in nursing because of my loyalty to this profession	3.03	1.02	0.70

Deep structure usage	I use an electronic medical records system to understand patients' records (e.g., medical history and family history)	4.23	0.73	0.95
	I use an electronic medical records system to understand patients' health trajectories	4.24	0.68	0.96
	I use an electronic medical records system to understand patients' drug interactions and considerations	4.17	0.74	0.88
	I use an electronic medical records system to help me with care evaluation	4.13	0.73	0.81
	I use an electronic medical records system to plan the care process	4.07	0.78	0.75
	I use an electronic medical records system to guide me in arranging work shifts	4.22	0.67	0.84

Organizational turnover intention	I plan to work in this hospital for a while⁣^∗^	2.98	1.30	0.69
	I plan to be working in this hospital 5 years from now⁣^∗^	3.15	1.18	0.55
	I plan to work in this hospital until retirement⁣^∗^	3.38	1.21	0.71

Abbreviations: *λ*, indicator loading; *M*, mean; SD, standard deviation.

⁣^∗^Denotes reversely coded items.

**Table 3:** Profile summary of the study participants.

Demographic	Category	Cases	Percentage
Gender	Female	410	98.3
	Male	7	1.7

Age	≥ 20 and < 30 years old	188	45.1
	≥ 30 and < 40 years old	124	29.7
	≥ 40 and < 50 years old	90	21.6
	≥ 50 years old	14	3.4
	Missing	1	0.2

Education	Junior college	57	13.7
	University or college	349	83.7
	Graduate institute or above	9	2.1
	Missing	2	0.5

**Table 4:** Correlations among the study constructs.

	1	2	3	4	5
1. Affective commitment	**0.73**				
2. Continuance commitment	0.10	**0.77**			
3. Normative commitment	0.43	0.22	**0.69**		
4. Deep structure usage of electronic patient records	0.34	0.12	0.14	**0.87**	
5. Organizational turnover intention	−0.30	−0.35	−0.19	−0.18	**0.66**

Mean	3.90	3.27	2.92	4.18	3.17
Standard deviation	0.63	0.85	0.77	0.64	1.05
Cronbach's *α*	0.84	0.83	0.76	0.94	0.82
Composite reliability	0.87	0.85	0.78	0.95	0.69

*Note:* All the numbers have *p* values < 0.05. The bold numbers on the diagonal are the square roots of the average variance extracted (AVE) values.

**Table 5:** Sources of organizational turnover intention.

	M1	M2	M3
Block 1			
Gender	−0.03	−0.07	−0.04
Age	−0.32⁣^∗∗^	−0.22⁣^∗∗^	−0.21⁣^∗∗^
Education	0.03	0.07	0.07
Block 2			
Affective commitment		−0.24⁣^∗∗^	−0.24⁣^∗∗^
Continuance commitment		−0.27⁣^∗∗^	−0.31⁣^∗∗^
Normative commitment		0.03	0.04
Deep structure usage of electronic patient records		−0.08	−0.07
Block 3			
Deep structure usage of electronic patient records × affective commitment			−0.09⁣^∗^
Deep structure usage of electronic patient records × continuance commitment			0.10⁣^∗^
Deep structure usage of electronic patient records × normative commitment			−0.03
Adjusted *R*^2^	0.11⁣^∗∗^	0.24⁣^∗∗^	0.25⁣^∗∗^

*Note:* Coefficients are standardized regression coefficients.

⁣^∗^Denotes *p* < 0.05; ⁣^∗∗^Denotes *p* < 0.01.

## Data Availability

The authors do not have the right to share the data, due to IRB restrictions.
